# The modulating role of uniaxial straining in the IL-1β and TGF-β mediated inflammatory response of human primary ligamentocytes

**DOI:** 10.3389/fbioe.2024.1469238

**Published:** 2024-12-10

**Authors:** Johannes Heidenberger, Raphael Hangel, Eva I. Reihs, Jonathan Strauss, Petra Liskova, Jürgen Alphonsus, Cornelia Brunner, Kevin Döring, Iris Gerner, Florien Jenner, Reinhard Windhager, Stefan Toegel, Mario Rothbauer

**Affiliations:** ^1^ Karl Chiari Lab for Orthopaedic Biology, Department of Orthopedics and Trauma Surgery, Medical University of Vienna, Vienna, Austria; ^2^ Institute of Applied Synthetic Chemistry, Faculty of Technical Chemistry, Technische Universitaet Wien, Vienna, Austria; ^3^ Division of Orthopedics, Department of Orthopedics and Trauma Surgery, Medical University of Vienna, Vienna, Austria; ^4^ Veterinary Tissue Engineering and Regenerative Medicine Vienna (VETERM), Equine Surgery Unit, University of Veterinary Medicine Vienna, Vienna, Austria; ^5^ Ludwig Boltzmann Institute of Arthritis and Rehabilitation, Vienna, Austria

**Keywords:** mechanical loading, ligamentocytes, inflammation, fibrosis, *in vitro* modelling

## Abstract

Biomechanical (over-)stimulation, in addition to inflammatory and fibrotic stimuli, severely impacts the *anterior cruciate ligament (ACL)* biology, contributing to the overall chronic nature of desmopathy. A major challenge has been the lack of representative two-dimensional (2D) *in vitro* models mimicking inflammatory processes in the presence of dynamic mechanical strain, both being crucial for ligament homeostasis. Physiological levels of strain exert anti-inflammatory effects, while excessive strain can facilitate inflammatory mechanisms. Adhering to the 3Rs (Replacement, Reduction and Refinement) principles of animal research, this study aims to investigate the role of a dynamic biomechanical *in vitro* environment on inflammatory mechanisms by combining a Flexcell culture system with primary human ligamentocytes for the study of ligament pathology. Primary ligamentocytes from OA patients were cultured under animal-free conditions with human platelet lysate, and exposed to either IL-1β or TGF-β3 to simulate different inflammatory microenvironments. Cells were subjected to different magnitudes of mechanical strain. Results showed that cells aligned along the force axis under strain. This study highlights the critical role of the mechanical microenvironment in modulating inflammatory and fibrotic cellular responses in ligamentocyte pathology, providing valuable insights into the complex interplay between biomechanical stimuli and cytokine signaling. These findings not only advance our understanding of ligament biology but also can pave the way for the development of more targeted therapeutic strategies for ligament injuries and diseases, potentially improving patient outcomes in orthopedic medicine.

## 1 Introduction

Ligaments are critical components of the human musculoskeletal system, playing a fundamental role in maintaining joint stability and functionality. Composed primarily of collagen fibers (90% collagen type I and 10% collagen type III), ligaments connect bones, in turn regulating movement, and provide essential stability, particularly in complex joints such as the knee (Riechert et al., 2001; Thorpe et al., 2010). These fibers are organized within an extracellular matrix (ECM), constructed and remodeled by specialized fibroblast cells known as ligamentocytes. Despite of their pivotal role in maintaining and repairing ligament tissue, the research on their behaviour under dynamic culture conditions remains very limited, in contrast to tenocytes (Provenzano et al., 2002). Nonetheless, understanding the cellular dynamics of ligaments is crucial for elucidating their fascinating mechanobiological niche in health and disease (Hasegawa et al., 2013; Schulze-Tanzil, 2019; Tozer and Duprez, 2005). Desmopathy is a debilitating condition and may play a particular role in joint destabilization and degeneration when affecting the anterior and posterior cruciate ligaments (ACL/PCL), which are responsible to stabilize the knee joint. Most research to date focuses on full or partial ligament ruptures in the context of tissue engineering and regenerative medicine, leaving the synergistic effects of mechanical loading in the presence of a pro-inflammatory ligament tissue response unexplored. Even though ‘normal’ strain is considered to maintain homeostasis and to have anti-inflammatory effects on tissues including tendons and ligaments, previous reports indicate that super-physiological strain can contribute to a pro-inflammatory microenvironment (Agarwal et al., 2003).

Within this complex cellular landscape, chronic inflammation, also known as desmopathy, emerges as a major contributing factor to ligament pathologies, second only to traumatic injuries, and is often driven by factors such as interleukin-1β (IL-1β). IL-1β is a pro-inflammatory cytokine that promotes the expression of other inflammatory mediators, exacerbating persistent tissue inflammation and damage that impairs natural wound healing process (Chamberlain et al., 2014; Xie et al., 2013b). Notably, IL-1β increases expression of key fibrotic mediator TGF-β1 in tenocytes (Morita et al., 2019). IL-6 is another crucial cytokine involved in the inflammatory response and tissue repair in ligaments. It is recognized not only as a pro-inflammatory marker but also as anti-inflammatory mediator. Its functions include stimulating the secretion of the anti-inflammatory cytokine IL-10 and inhibiting TNF-α (Docherty et al., 2022). As a central regulator of tissue repair, TGF-β plays multiple roles in wound healing and fibrosis. TGF-β modulates the synthesis of ECM components and influences the balance between fibrosis and tissue regeneration, critical for maintaining ligament integrity (Wang et al., 2011; Xie et al., 2013a). While TGF-β1 is typically induced early in tissue injury, all three TGF-β isoforms exhibit profibrotic actions *in vitro*, activating similar signaling pathways and stimulating ECM protein synthesis (Frangogiannis, 2020).

While these cytokines play a major role in desmopathy, it is also of high importance for ligamentocytes to be able to sense mechanical forces acting on them, to ensure their physiological development and tissue homeostasis. Mechanosensing, which constitutes a fundamental aspect of ligament physiology involves stretch-activated ion channels, such as PIEZO1 to regulate ECM stiffness, and hence influences overall tissue stiffness (Passini et al., 2021). Consequently, ligamentocytes require mechanical stimuli to maintain overall tissue integrity similar to other musculoskeletal cell types such as myocytes, tenocytes or chondrocytes (Benhardt and Cosgriff-Hernandez, 2009). PIEZO1 channels are sensitive to mechanical forces, and their (over- or under-) activation can trigger also disease-relevant signaling pathways like the NF-κB pathway as master regulator that modulates cellular processes including proliferation, differentiation, and cell-matrix interactions (Jin et al., 2015). Interestingly, although stimulation of PIEZO1 may inhibit inflammation but can also induce fibrosis via activation of TGF-β/Smad and p38-MAPK signaling (Morita et al., 2019; Xu et al., 2023). To investigate the effect of a mechanical microenvironment on ligamentocytes, *in vitro* modeling approaches utilizing mechanically stimulated 2D and 3D cell cultures (e.g., the FlexCell system or the FlexiCell system) enable the investigation of strain profiles to impact cell (patho-) physiology (Sun et al., 2016). A better understanding of the intricate mechanisms of the mechanical and biochemical cell microenvironment in ligament physiology will be paramount to advance ligament tissue engineering and disease modeling in a variety of musculoskeletal pathologies including desmopathies (Rothbauer et al., 2020; Rothbauer et al., 2021).

Current *in vitro* models often lack an inflammatory component when investigating strain. Therefore, in this study we set out to investigate the interplay between mechanical stress, interleukin-mediated inflammation, and TGF-β-mediated fibroblast activation and provide valuable preliminary insights into how pathological tissue responses are modulated by a mechanically loaded cell microenvironment. Initially, the effect of hypoxia, cell seeding density and 10% normal strain loading was investigated for culture optimizations under animal-free conditions using human platelet lysate (hPL). Primary ligamentocytes from four patients were then cultured in the presence of either IL-1β (1 ng/mL) to induce pro-inflammatory conditions or TGF-β3 for stimulation of a more pro-fibrotic microenvironment. Cells were strained at 0.5 Hz for 48 h at 10% (normal) and 21% (supra) elongation, then imaged and analyzed by RT-qPCR. We investigated the influence of super-physiological strain on ligamentocyte responses in both basal, pro-fibrotic and pro-inflammatory environments using the FX-5000 Tension System (FlexCell) on primary human ligament cell cultures to investigate its modulating effect on cell orientation as well as pro-inflammatory response.

## 2 Methods

### 2.1 Cell culture

Cruciate ligament tissue samples of arthroplasty patients (total of n = 14 of mixed sex) with a patient age between 55 and 84 were harvested with informed patient consent and cryogenic stocks were used from the ViBiMed biobank (EK-No. 1822/2017 and 1268/2024) for all *in vitro* experiments with approval by the ethics committee of the Medical University of Vienna. Primary ligament cells were cultured in DMEM containing 4.5 g/L D-Glucose, 25 mM HEPES, further supplemented with human platelet lysate (ELAREM Perform-FD PE21012, PL Bioscience) and Antibiotic-Antimycotic (Gibco). The cells were cultured at 37°C under either normoxic (21% O_2_ and 5% CO_2_) or hypoxic (5% O_2_ and 5% CO_2_) conditions and subpassaged at 90% confluence using animal-free TripLe Express solution (Gibco, no phenol red, 12604013). To induce a pro-inflammatory state in the FlexCell culture for 48 h (see Section 2.3), complete culture medium was supplemented with 1 ng/mL IL-1β (Human Recombinant IL-1β, in PBS with 1% human or bovine serum albumin, BLD, Biozym Scientific GmbH). Similarly, complete medium was supplemented with 1 ng/mL of TGF-β3 (Recombinant Human TGF-beta three protein, R&D SYSTEMS, Bio-techne) reconstituted in HCL containing 1 mg/mL human serum albumin according to the manufacturer’s instructions. These IL-1β and TGF-β3 concentrations were chosen as they were previously shown to stimulate the cells in a significant manner (Farhat et al., 2012; Zhang et al., 2013).

### 2.2 RT-qPCR preparations and analysis

RNA concentration and purity was analyzed from a 1 µL sample using a *Nanodrop 2000 system* (ThermoFisher Scientific). RT-qPCR including mRNA isolation and cDNA synthesis was performed as previously described (Killinger et al., 2023). RNA was isolated using 350 μL of RNA Lysis buffer (innuPREP RNA Mini Kit 2.0, AJ Innuscreen GmbH) according to the manufacturer’s protocol for cell preparations. In brief, after an incubation period for at least 3 min at room temperature, the lysate was transferred to the spin filter column D (Spin Filter D, innuPREP RNA Mini Kit 2.0, AJ Innuscreen GmbH) and centrifuged for 2 min at 11,000 rpm. The filtrate was collected, and 350 μL of 70% ethanol was added. After transferring the mixture into spin filter column R (Spin Filter R, innuPREP RNA Mini Kit 2.0, AJ Innuscreen GmbH) and centrifuged again at 11,000 rpm for 1 min, the flow-through was discarded. Now 500 μL of high salt solution (Washing Solution HS (conc.), innuPREP RNA Mini Kit 2.0, AJ Innuscreen GmbH) was added to the column and centrifuged again at 11,000 rpm for 1 min. Afterwards, the flow-through was discarded, 700 μL of low salt solution (Washing Solution LS (conc.), innuPREP RNA Mini Kit 2.0, AJ Innuscreen GmbH) were added and again spun at 11,000 rpm. The flow-through was discarded again, and the column was dryed by spinning it a at 11,000 rpm for 3 min. The final flow-through was discarded, and the column was placed in prepared Eppendorf tubes. Then 40 μL of molecular grade water was used to extract the RNA from the column. cDNA was synthesized using the High-capacity cDNA Reverse Transcription Kit (Applied Biosystems™, ThermoFisher Scientific) according to the manufacturer’s instructions using a 5,331 Mastercycler Gradient Terminal Cycler (Eppendorf). The cDNA was diluted in a 1:5 ratio with molecular grade water, and 1 μL of diluted cDNA was mixed with 10 μL of PowerTrack SYBER Green master mix (ThermoFisher Scientific), 2.5 μL of forward primer, 2.5 μL of backward primer and 4 μL of molecular grade water (for human primer sequences see Table 1).

**TABLE 1:** Human primer sequences.

Gene	Forward primer	Reverse primer
COL1A1	CAC​TGG​TGA​TGC​TGG​TCC​TG	CGA​GGT​CAC​GGT​CAC​GAA​C
COL3A1	GAA​AGA​GGA​TCT​GAG​GGC​TCC	AAA​CCG​CCA​GCT​TTT​TCA​CC
MMP1	TCT​AGA​AAC​ACA​AGA​GCA​AGA​TGT​G	GCG​TGT​AAT​TTT​CAA​TCC​TGT​AGG​T
PIEZO1	TCG​GAC​CAG​TCT​GTG​GTC​AT	TCG​ATG​ACC​CAC​CAT​TCG​AG
SDHA	TGG​GAA​CAA​GAG​GGC​ATC​TG	CCA​CCA​CTG​CAT​CAA​ATT​CAT​G
IL6	ATA​GGA​CTG​GAG​ATG​TCT​GAG​G	AGG​ACA​ACT​GGA​CCG​AAG​G

### 2.3 Uniaxial loading of 2D ligamentocytes using a FlexCell tension system

To apply strain on 2D ligamentocyte cultures, the FX-5000 Tension System (FlexCell International Corporation) was used. After seeding 65,000-300,000 cells on a 6-well BioFlex Plate (FlexCell International Corporation), they were incubated for 48 h and placed into the tension system. One of two training regimes was used for the experiments, either the normal strain or overstrain loading. Both regimes were loaded at the same frequency at 0.5 Hz and duration of 48 h, differing only in the strain amplitude corresponding to either 10% or 21% estimated elongation, respectively.

### 2.4 Statistical analysis

Statistical analysis of gene expression data was performed using GraphPad Prism 10.2.0. Specifically, the Wilcoxon signed-rank test was used to calculate the significance for data of two groups (*P < 0.05, **P < 0.05). The one-way ANOVA with Dunnett’s multiple comparisons test was conducted for analyzing data among three groups. An α of 0.05 was chosen and any P values <0.05 were considered statistically significant (*P < 0.05, **P < 0.01, ***P < 0.001, ****P < 0.0001).

## 3 Results and discussions

### 3.1 Impact of cultivation protocol on ligamentocyte biology

**FIGURE 1:**
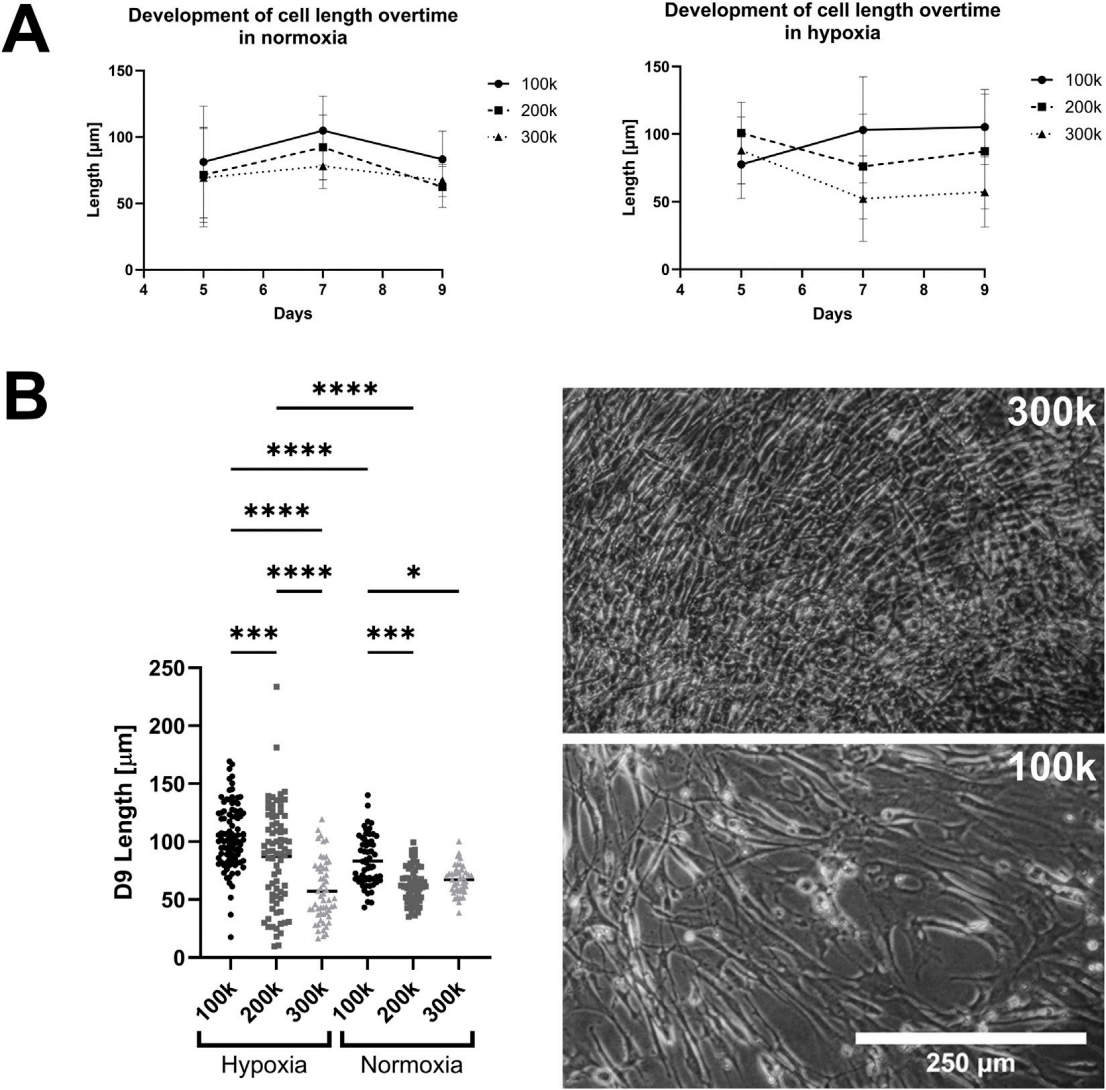
Effect of oxygenation and seeding conditions on ligamentocyte morphology. **(A)** Mean cell length of 2D ligamentocyte cultures at initial cell seeding of 100 - 300 k cells per well in normoxic and hypoxic conditions over the culturing period. **(B)** Depiction of the significant differences in cell length between different seeding densities in normoxia and hypoxia. Representative images of ligamentocyte cultures at 300k (top) and 100k (bottom) seeding densities are displayed on the right. Data are expressed as scatter plot with mean for measurements of 43–93 cells of biological duplicates. Significance was determined with a two-way ANOVA. (*P < 0.05, **P < 0.005, ***P < 0.0005, ****P < 0.0001).

To understand how hypoxia and seeding densities affect patient-derived anterior cruciate ligament (ACL) cell morphologies, we initially cultured three different cell numbers over a 9-day period in normoxic (20%) as well as hypoxic (5%) conditions. Ligamentocyte length was monitored up to day 9 and gene expression was determined for HIF1A, COL1A1 and COL3A1 as ligament matrix-related target genes. Figure 1 shows the change of cell length over time in normoxic and hypoxic conditions. In the normoxic condition (Figure 1A), the mean cell length was slightly elongated from day 5 to day 7 by about 20 µm for wells seeded with 100 k and 200 k cell density (*p* > 0.05) with the lowest cell length at the highest cell density of 300 k cells per well due to cell over-crowding. Similar outcomes were observed for another 4 days in hypoxic culture. When investigating the same cultures in more detail at the single cell level under hypoxic culture conditions (Figure 1B), a pronounced decrease of cell length by approx. 18 μm and 48 µm over the course of 9 days was visible for the 200 k and 300 k seeding densities compared to 100 k initial seeding density (*p* < 0.0005 and *p* < 0.0001), respectively. For the lowest seeding density of 100 k cells per well, hypoxic conditions showed a significant increase in ligamentocyte length from 83 ± 21 to 105 ± 25 µm within 9 days of static cultivation (*p* < 0.0001), as shown in the representative phase contrast images (Figure 1B right image panel). Overall, data suggests that hypoxic conditions in combination with lower surface coverage results in a desired elongated ligamentocyte morphology for more physiological cell culture since they display longer protrusions, which resemble more the natural *spindle-shaped (fusiform) state* inside ligaments. Our data aligns well with previous studies confirming that hypoxic conditions can be beneficial for culturing tenocytes and ligamentocytes, but can also have detrimental cellular effects due to overcrowding (Rhatomy et al., 2021).

**FIGURE 2:**
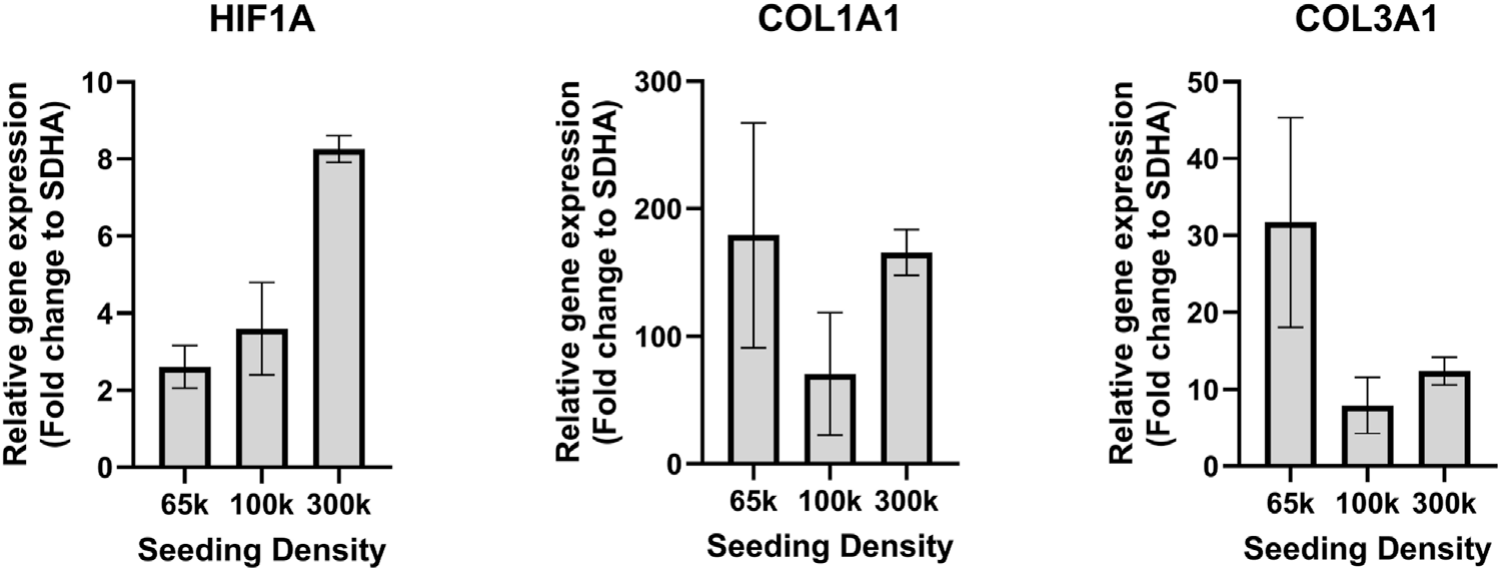
Gene expression of ligamentocytes in hypoxic condition. HIF1A, COL1A1 and COL3A1 gene expression in a hypoxic environment for different cell seeding densities in a BioFlex 2D cell culture including 65 k (n = 8 biological replicates), 100 k (n = 4) and 300 k (n = 2).

To investigate the beneficial effect of hypoxia and sparse cell seeding, RT-qPCR analysis was performed for HIF1A, COL1A1 and COL3A1 target genes to elaborate a bit more on the role of hypoxia over normoxia on ligamentocyte physiology in more detail for 65 k, 100 k and 300 k initial cell seeding densities. Figure 2 confirms that HIF1A expression was significantly higher (3.1-fold change) for the highest seeding density of 300 k compared to the lowest seeding density of 65 k that we hypothesized to further improve gene expression outcomes. COL1A1 expression was comparable between culture conditions. Furthermore, we investigated the change of COL3A1 mRNA expression (Figure 2 right graph), which we expected to increase in the case of pro-fibrotic cell responses (Nichols et al., 2019; Sun et al., 2020). Figure 2C shows that ligamentocytes at 65 k cells per well led to an approximate 2.9-fold higher expression of COL3A1 compared to the 300k seeding density. Previous studies on mesenchymal stem cells, which are considered to be anti-fibrotic stromal cells, discussed increased COL3A1 expression levels in a physiological hypoxic environment compared to normoxia (Yu et al., 2016). Because the primary goal of this culture optimization was to identify a physiological condition for ligamentocytes, all subsequent experiments investigating the impact of mechanical loading were conducted using the optimized culture protocol using a seeding density of 65 k cells per well under 5% hypoxic conditions.

### 3.2 Effect of strain application on ligamentocyte orientation

**FIGURE 3:**
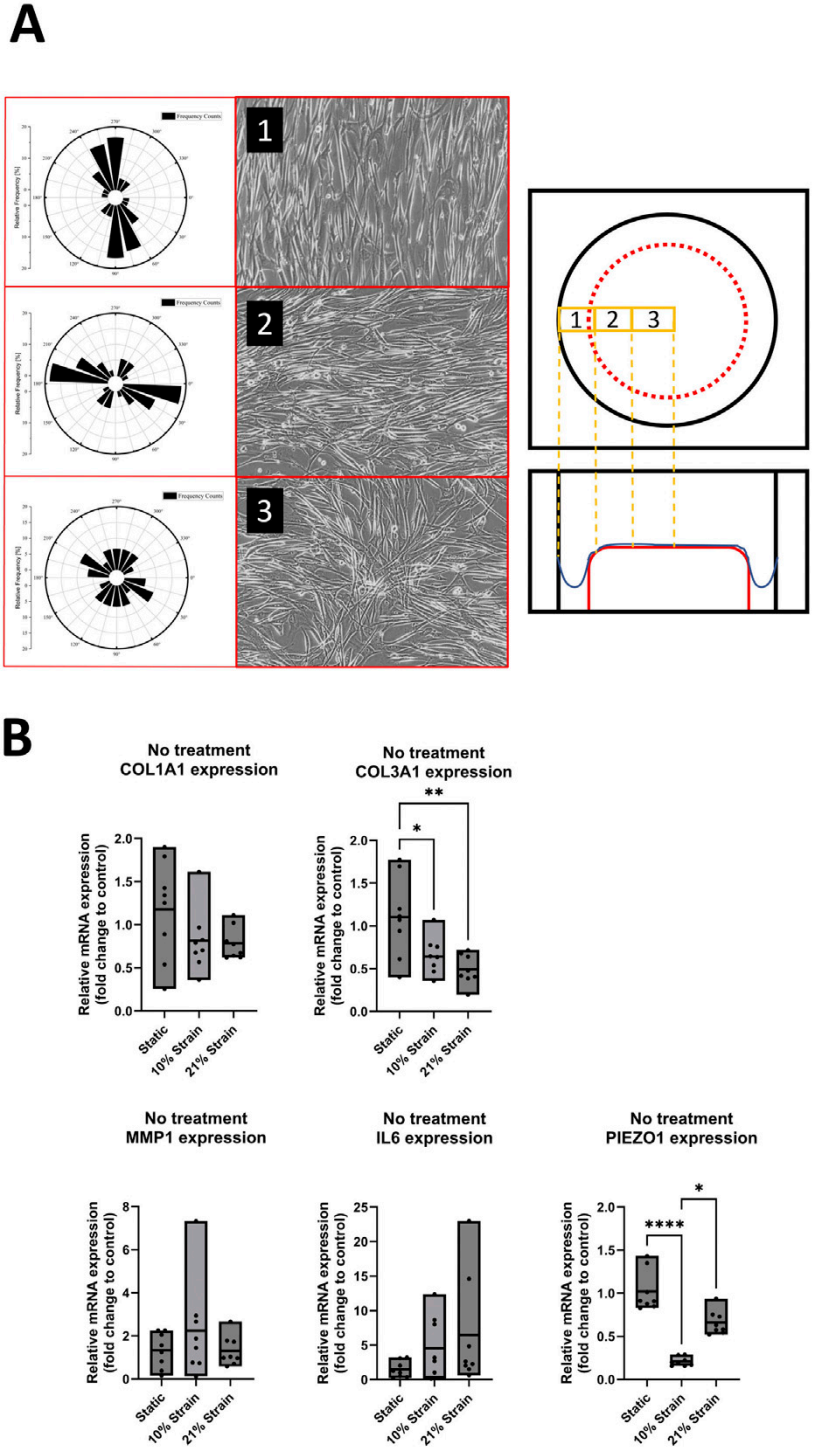
Ligamentocyte cell alignment and gene expression in flex cell culture with 10% and 21% strain and with IL-1β or TGF-β treatment. **(A)** The polar graph and brightfield images from three different zones (1: Border, 2: Transition, 3: Center) inside a flex cell well show the cell alignment for 10% strain. **(B)** Ligamentocytes were trained for 48 h in a flexcell system without any treatment. Different amounts of strain were applied to the cells (10%, 21%) and gene expression of COL1A1, COL3A1, MMP1, IL6 and PIEZO1 was analyzed with qPCR. The data is expressed as fold change to the mean of the untreated static control. n = 8 biological replicates. Significance was determined with a one-way ANOVA with Dunnett’s multiple comparisons test. (*P < 0.05, **P < 0.01, ***P < 0.001, ****P < 0.0001).

To investigate the effect of uniaxial mechanical strain on cell orientation along the strain direction, human ligamentocyte cultures were loaded mechanically under hypoxia using BioFlex 6 well plates for up to 7 days. Brightfield images were taken of three different locations in the well and the orientation of the cells was measured with ImageJ in relation to the horizontal axis. Additionally, expression for COL1A1, COL3A1, MMP1, IL6 and PIEZO1 was analyzed in more detail. The polar angle analyses of Figure 3A indicate the orientation of the cells in the three zones of a Flexcell well, including Border (1), Transition (2), and Centre regions (3), with representative brightfield images of ligamentocytes on the right showing the orientation during image analysis. Overall, data suggests that even though all cells may be stretched within the FlexCell system, only approx. 33% of the area is loaded along the strain axis, with cells of the transition zone showing an orientation perpendicular to the loading direction. Interestingly, cells located at the central region exhibited a random orientation, without alignment along the strain axis. Our data confirms this heterogenous loading of the FlexCell system indicated in previous studies where the structure of the surface topography itself led to unwanted orientation in the case of fibroblasts (Loesberg et al., 2005), which are known to be more responsive to surface topographies than other cell types such as endothelial or smooth muscle cells (Biela et al., 2009). Figure 3B shows the effect of 10% and 21% loading impact on ligamentocyte physiology. While COL1A1 showed no significant response towards both loading conditions (*p* > 0.05), COL3A1 gene expression was attenuated directly proportional to the straining strength to 0.7 ± 0.2 fold (*p* < 0.05) and 0.5 ± 0.17 fold (*p* < 0.01), respectively. Interestingly, neither 10% nor 21% loading showed a response for pro-inflammatory genes MMP1 and IL6. Lastly, 10% loading significantly decreased PIEZO 1 expression to 0.2 ± 0.05 fold (*p* < 0.0001).

### 3.3 The modulating effect of mechanical (over)loading on a pro-inflammatory ligamentocyte environment

A proinflammatory microenvironment constitutes a common issue with ligament pathologies including both chronic, as well as acute traumatic injuries. While ruptures and other physical traumas are often the primary focus of research concerning ligaments, chronic inflammation and fibrotic tissue remodelling receive comparatively little attention. Ligaments require mechanical stimulation to function normally, which additionally can have an anti-inflammatory effect. However, the efficacy of this effect is highly dependent on the magnitude of the stimulation as low strain (3%–8% elongation) has been shown to have an anti-inflammatory effect on dental ligament cells, while high strain regimes (exceeding 15% elongation) yielded a pro-inflammatory response. To elaborate on these effects for ACL-derived primary ligamentocytes, we subjected patient cells to either normal strain (10% straining) or an extreme overstrain training regimen (21%) under hypoxic conditions. As our primary focus was to establish a pathological cell microenvironment, we investigated the physical loading effects for a 48 h period in parallel to treatment regimens with IL-1β and/or TGF-β3, which are commonly used inflammatory and fibrosis mediators. Initially, the responsiveness of the primary ligamentocytes towards a pro-inflammatory stimulus such as IL-1β was investigated to record a baseline for static inflamed cells.

**FIGURE 4:**
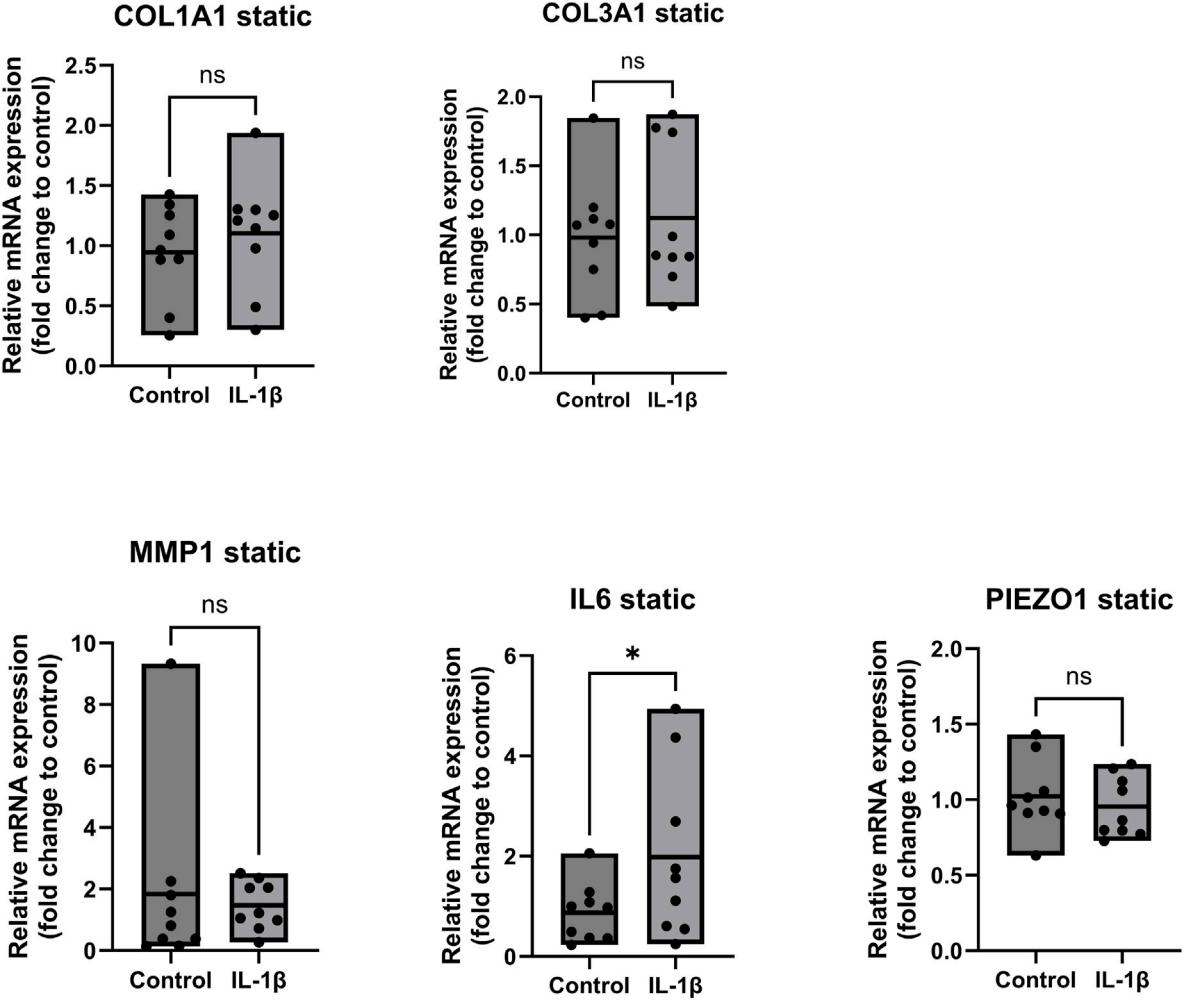
Gene expression of ligamentocytes in a static Flexcell culture with and without IL-1 treatment. Ligamentocytes were treated with IL-1β (1 ng/mL) for 48 h in a flexcell system. Gene expression of COL1A1, COL3A1, MMP1, IL6 and PIEZO1 was analyzed with qPCR. The data is expressed as fold change to the mean of the untreated static control. n = 9 biological replicates. Significance was determined with a Wilcoxon test. (*P < 0.05, **P < 0.05).

Figure 4 shows that the gene expression of COL1A1, COL3A1, MMP1 and PIEZO1 exhibit no significant trend in static samples treated with IL-1β compared to the untreated control. However, we observed a significant upregulation by 2-fold (*p* < 0.05) of IL6 in the group treated with IL-1β, confirming the anticipated inflammatory activation of the tissue-derived cells. This well-documented pro-inflammatory effect of IL-1β has been extensively described in numerous studies, not only in human models but also in equine patients (Beaumont et al., 2024; Cahill and Rogers, 2008; Chen et al., 2005; Song et al., 2019).

**FIGURE 5:**
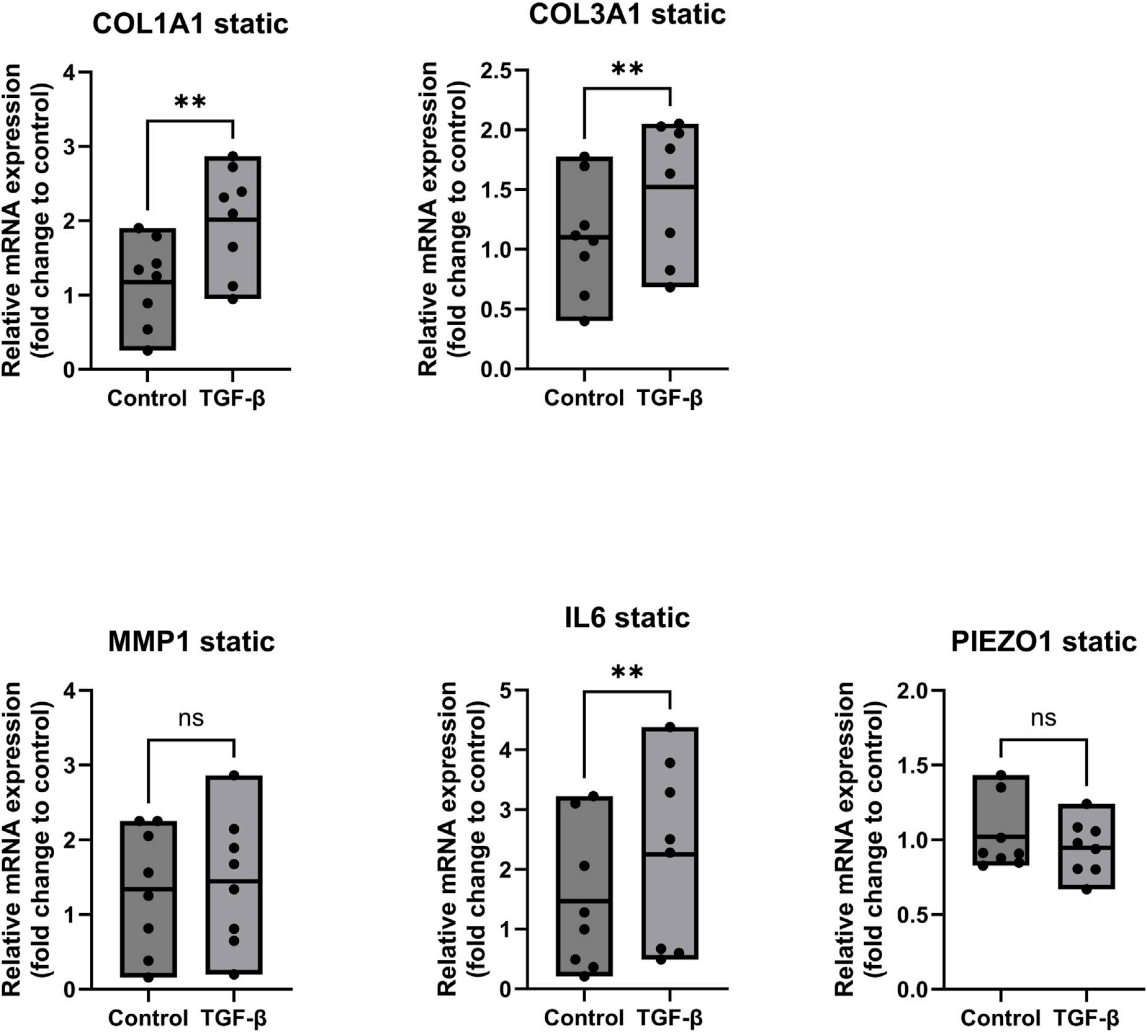
Gene expression of ligamentocytes in a static Flexcell culture with and without TGF- β treatment. Ligamentocytes were treated with TGF-β (1 ng/mL) for 48 h in a flexcell system. Gene expression of COL1A1, COL3A1, MMP1, IL6 and PIEZO1 was analyzed with qPCR. The data is expressed as fold change to the mean of the untreated static control. n = 8 biological replicates. Significance was determined with a Wilcoxon test. (*P < 0.05, **P < 0.05).

Figure 5 depicts the gene expression of ligamentocytes treated with TGF-β in static conditions. We observed a significant increase in gene expression of COL1A1 by 2-fold (*p* < 0.05), COL3A1 by 1.5-fold (*p* < 0.05) and IL6 by about 2.2-fold (*p* < 0.05) for the TGF-β treated ligamentocytes. These findings align with our understanding of TGF-β as a profibrotic mediator, as evidenced by the upregulation of collagen genes COL1A1 and COL3A1, along with a simultaneous increase in IL6 expression, highlighting its dual role in promoting extracellular matrix production and inflammatory signalling. Similar findings were made by Altmann et al., who found upregulated collagen type 1 and 3 in tendon progenitor cells when treated with IL6 (Altmann et al., 2023). Furthermore, a study from the University of Oklahoma showed that IL6 knockout fibroblasts (dermal) increased their expression of TGF-β1 when treated with recombinant IL6, which hints at a positive feedback loop between IL6 and TGF-β (Luckett-Chastain and Gallucci, 2009). Both these studies suggest that the interplay between TGF-β and IL6 has a prominent role on collagen expression in fibroblasts, which is undermined by our findings in Figure 5.

**FIGURE 6:**
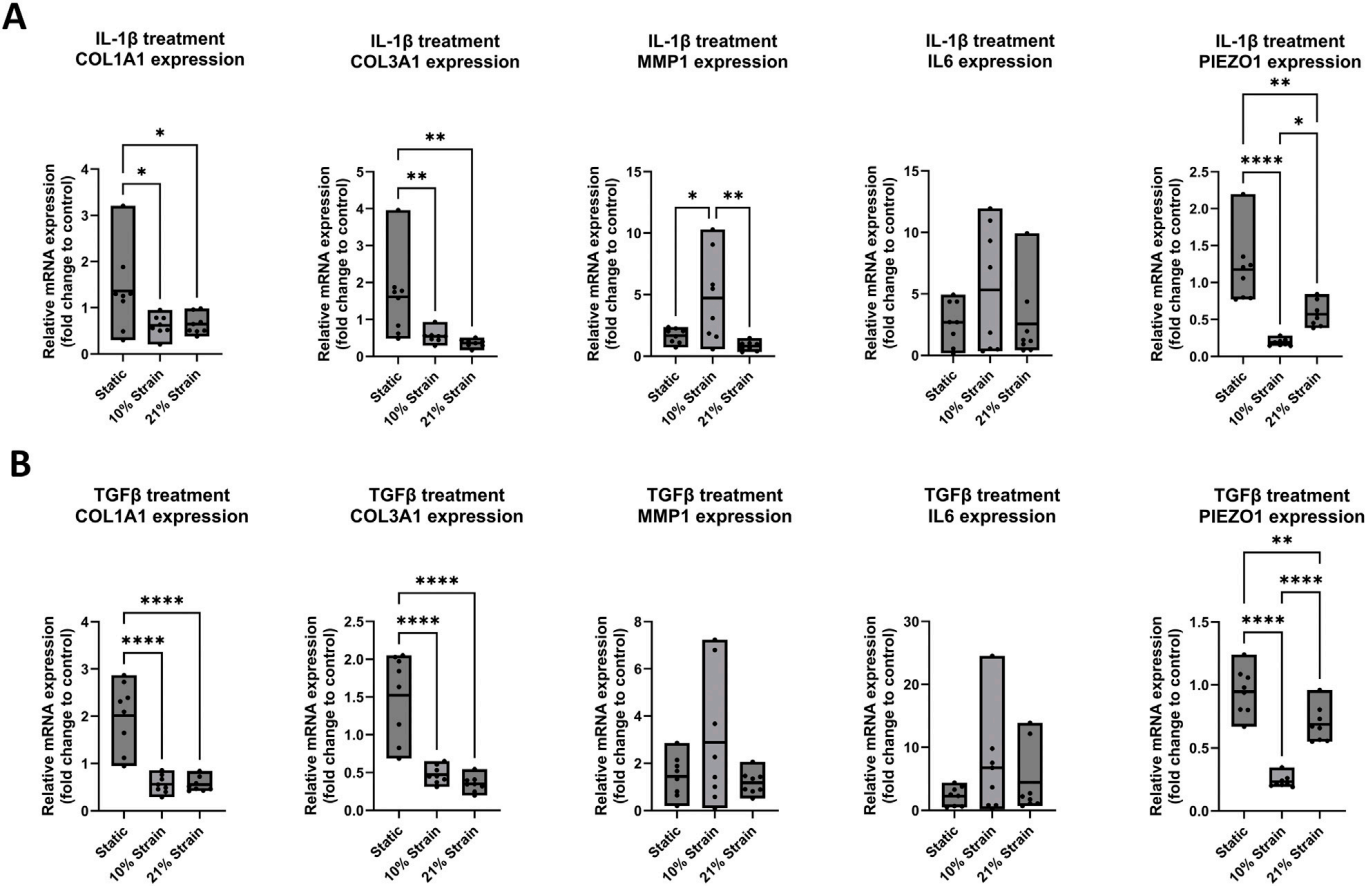
Gene expression of ligamentocytes in a Flexcell culture with 10% and 21% strain and with IL-1β or TGF-β treatment. Ligamentocytes were trained for 48 h in a flexcell system with **(A)** IL-1β (1 ng/mL) **(B)** and with TGF-β (1 ng/mL) treatment. Different amounts of strain were applied to the cells (10%, 21%) and gene expression of COL1A1, COL3A1, MMP1, IL6 and PIEZO1 was analyzed with qPCR. The data is expressed as fold change to the mean of the untreated static control. n = 8 biological replicates. Significance was determined with a one-way ANOVA with Dunnett’s multiple comparisons test. (*P < 0.05, **P < 0.01, ***P < 0.001, ****P < 0.0001).

**FIGURE 7:**
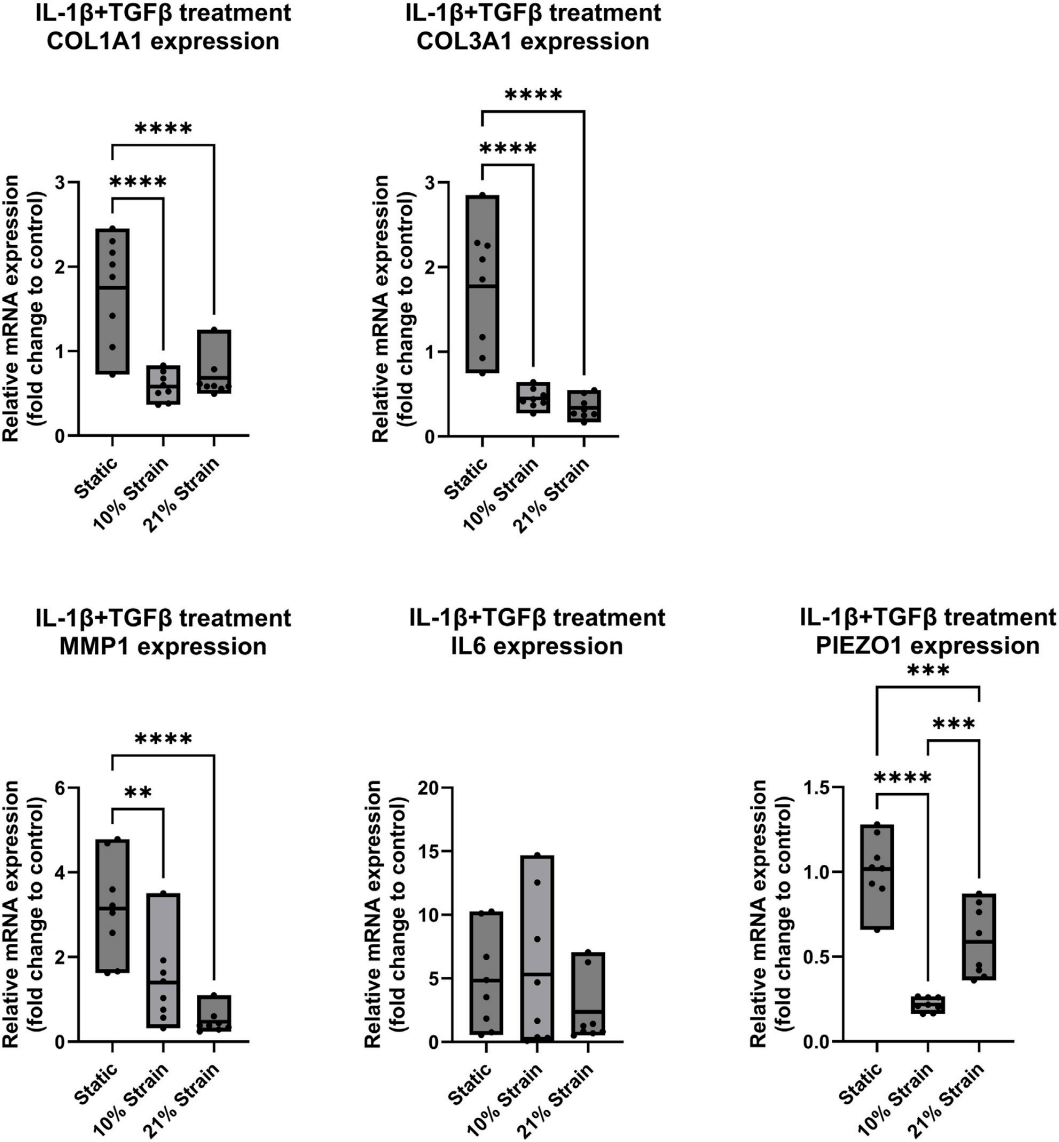
Gene expression of ligamentocytes in a Flexcell culture with 10% and 21% strain and IL-1β and TGF-β3 treatment. Ligamentocytes were trained and treated together with TGF-β (1 ng/mL) and IL-1β (1 ng/mL) for 48 h in a flexcell system. Different amounts of strain were applied to the cells (10%, 21%) and gene expression of COL1A1, COL3A1, MMP1, IL6 and PIEZO1 was analyzed with qPCR. The data is expressed as fold change to the mean of the untreated static control. n = 8 biological replicates. Significance was determined with a one-way ANOVA with Dunnett’s multiple comparisons test. (*P < 0.05, **P < 0.01, ***P < 0.001, ****P < 0.0001).

In a final set of experiments (Figures 6, 7), the response of ligamentocytes subjected to either 10% or 21% strain along with a single or combined treatment with IL-1β and TGF-β3 was investigated and compared to static results (see also Figure 3) to look into potential modulatory effects of loading in the context of a strong inflammatory cell microenvironment. Regarding the matrix markers COL1A1 and COL3A1 in Figures 6, 7 we found a significant downregulation in gene expression for both markers in all treatment groups when any amount of strain was applied to the cells. Under no treatment, COL3A1 expression decreased by 0.7-fold at 10% strain (*p* < 0.05) and 0.5-fold at 21% strain (*p* < 0.01); with IL-1β treatment, COL1A1 expression was reduced by 0.5-fold (*p* < 0.05), while COL3A1 exhibited a 0.4- to 0.5-fold decrease (*p* < 0.01); under TGF-β treatment, COL1A1 expression decreased by 0.5-fold for both strains (*p* < 0.0001), and COL3A1 decreased by 0.5-fold at 10% strain and 0.4-fold at 21% strain (*p* < 0.0001); with combined treatment, COL1A1 decreased by 0.6-fold at 10% strain and 0.7-fold at 21% strain (*p* < 0.0001), while COL3A1 showed a 0.5-fold decrease at 10% strain and 0.4-fold at 21% strain (*p* < 0.0001). In a study from Sánchez-Sánchez et al., an experimental rat model was used to induce tendinopathy, revealing elevated COL1A1 and COL3A1 mRNA levels in the diseased rats compared to the controls (Sánchez-sánchez et al., 2020). These findings contrast with our results in ligamentocytes. While this rat model may not directly reflect human ligament pathology, it underscores the significant differences between *in vitro* and *in vivo* systems, tendon and ligament biology, and human versus animal models, highlighting the complexity of translating findings across these different research domains. Regarding inflammation markers MMP1 and IL6 we found a significant increase in MMP1 expression for 10% strain by 5-fold (*p* < 0.05) in the groups treated with IL-1β (Figure 6A). On the other hand, we saw a significant decrease by 1.5-fold (*p* < 0.01) for 10% strain and 0.5-fold (*p* < 0.0001) for 21% strain in Figure 7 for the combined treatment group (IL-1β + TGF-β). These results suggest that strain application can lower the expression of MMP1 in an inflamed environment if TGF-β is present and thereby potentially mitigate tissue degradation by MMP1. IL6 on the other hand seemed rather unaffected by the strain application. In Figure 6A, B a slight trend towards higher IL6 expression is visible if strain was applied but this change was not significant. Interestingly, expressions of PIEZO1 showed a similar pattern of downregulation in the untreated control compared to the IL-1β and TGF-β and co-treated groups. In all groups we found that PIEZO1 was strongly downregulated in the 10% strain setting by 0.25-fold (*p* < 0.0001) and slightly downregulated in the 21% strain by 0.5-fold (*p* < 0.001) setting compared to the control. As PIEZO1 is responsible for mechanotransduction and adaptation of the cell to mechanical strain by tissue remodelling, we expected an increase in expression in response to the stretching of the cells. The results, however, do not align with this expectation, which could be due to a variety of factors, including time-point-specific expression, negative feedback for prolonged strain, or simply adaptation to the culture conditions. The fact that the downregulation was independent of the presence of cytokines leads to the hypothesis that there is a universal mechanism or pathway involving PIEZO1, which is independent of IL-1β and TGF-β signaling.

## 4 Conclusion

Overall, the current body of research on ligament inflammation is very thin, making direct comparisons to previous studies difficult. However, this also presents an opportunity to address this research gap. By investigating primary ligamentocytes under both healthy and diseased conditions, particularly in the context of mechanical strain, we can lay the foundation for a deeper understanding of ligament biology and its responses to pathological mechanical stimuli. In conclusion, we successfully optimized animal-free cultivation protocols for the study of primary tissue-derived ligamentocytes under hypoxia and with respect to seeding density which both influence gene expression and morphology of ACL-tissue derived cells. Furthermore, we study biomechanical processes using cell loading approaches. Our results suggest that mechanical loading attenuates matrix biosynthesis in inflamed and fibrotic conditions when uniaxial strain is applied. These findings emphasize the pivotal role of the mechanical microenvironment in shaping cellular responses to pro-inflammatory and pro-fibrotic mechanisms, providing new insights into ligamentocyte pathobiology.

## Data Availability

The raw data supporting the conclusions of this article will be made available by the authors, without undue reservation.
