# Supplementary material for: The modulating role of uniaxial straining in the IL-1β and TGF-β mediated inflammatory response of human primary ligamentocytes
Source: Front Bioeng Biotechnol. 2024 Dec 10;12:1469238. doi: 10.3389/fbioe.2024.1469238 (PMC11666359; doi:10.3389/fbioe.2024.1469238)
Supplement: Supplementary file 1 [file DataSheet1.PDF]

Supplementary Material

1 Supplementary Figures and Tables

1.1 Supplementary Figures

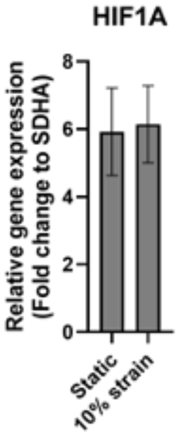

Supplementary Figure 1. Ligamentocyte gene expression in flex culture. Impact of dynamic 10% straining on the relative gene expression of HIF1A of patient-derived ligamentocytes. Data is expressed as fold-change relative to SDHA for n=2.

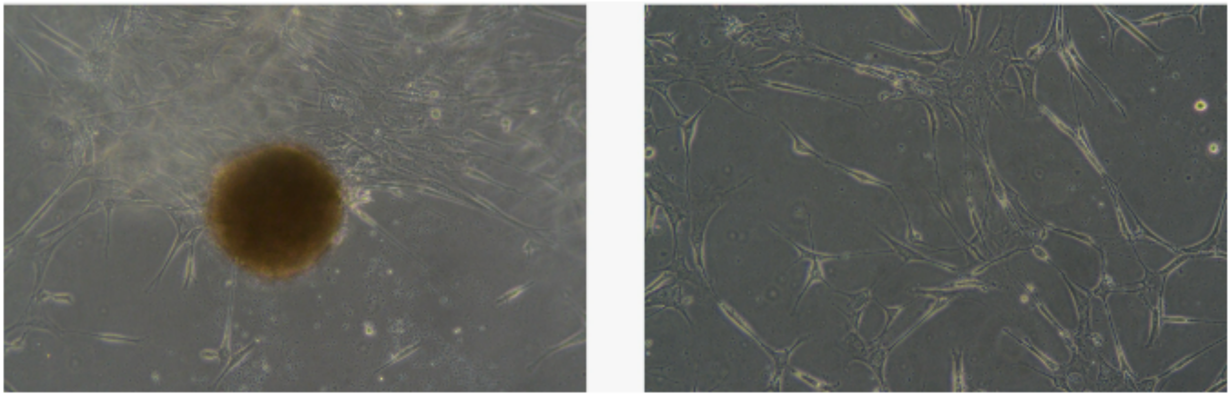

Supplementary Figure 2. Effect of overcrowding on cell adhesion in flex culture. Representative images of 300k (left image) and 65k (right image) after 48h of loading at 10% strain.
